# Proteomic datasets of three *Hermetia illucens* Larvae Forms (Fresh, Dried, and Protein concentrate)

**DOI:** 10.1016/j.dib.2026.112741

**Published:** 2026-03-31

**Authors:** Abdel-Moneim Eid Abdel-Moneim, Daniel Tomas, Valérie Labas, Bertrand Méda, Sophie Réhault-Godbert

**Affiliations:** a Biological Applications Department, Nuclear Research Center, Egyptian Atomic Energy Authority, 13759, Egypt; b PIXANIM, INRAE, Université de Tours, CHU de Tours, Nouzilly, 37380, France; INRAE, CNRS, Université de Tours, PRC, Nouzilly 37380, France; c INRAE, Université de Tours, BOA, Nouzilly, France

**Keywords:** Black soldier fly, Live, Dry, Proteomics, Feed alternative

## Abstract

Over the past few decades, black soldier fly (BSF) larvae have emerged as a promising alternative protein source for animal feed. In addition to providing essential amino acids that support animal performance, BSF larvae also contain certain bioactive components that may contribute to maintaining intestinal health of animals. However, it is assumed that depending on the process used to obtain these larvae, their composition in proteins and bioactive molecules may vary.

In this context, we analyzed the proteomes of live BSF larvae (BSFL), dehydrated BSFL, and a protein concentrate prepared from the dehydrated BSFL (all from the same commercial source). The larvae samples were frozen, freeze-dried, and ground before protein extraction. Proteins from each form were solubilized using a high concentration of sodium chloride and subsequently analyzed by mass spectrometry. The resulting protein lists were compared to identify shared proteins, sample-specific proteoforms, and gene identifiers. Venn diagrams showed that the three samples shared 888 proteins. Notably, live larvae exhibited the highest protein complexity, with 814 unique proteins, compared to none in dehydrated larvae, and 36 in the protein concentrate.

The compilation of all the data enabled the identification of 2232 unique gene identifiers. Gene ontology analysis using EnsemblMetazoa database revealed numerous proteins associated with metabolism (amino acids, lipids, carbohydrates), while 57 proteins were assigned to innate defense and detoxification processes. Approximately 30 antimicrobial proteins/peptides were identified (peptidoglycan-binding proteins, lysozymes, cecropins, defensins, and attacin-like peptides). These data highlight that BSF larvae are a natural source of potential bioactive compounds, including antimicrobial proteins and peptides.

Specifications Table

**Table:** 

Subject	Biology
Specific subject area	Proteome of black soldier fly larvae obtained after three technological processes
Type of data	Table, Figure, Processed data
Data collection	Three forms of black soldier fly larvae (live, dried, protein concentrate) were obtained from a commercial source, ground, and stored at −20 °C prior to protein solubilization in a buffer containing a high concentration of sodium chloride, followed by mechanical grinding. Four technical replicates were prepared for each condition. The samples were centrifuged, and the supernatants were filtered through a 0.45-µm membrane prior to quantification of protein concentration. The protein profile of each sample was analyzed by SDS-PAGE to assess technical reproducibility. The technical replicates were then pooled and fractionated onto a 10 % acrylamide-polyacrylamide gel for rapid electrophoresis, in order to obtain a single large band. After staining with Coomassie Blue, the three bands corresponding to each larval pool were excised, and subjected to in-gel digestion before analysis by nanoflow liquid chromatography tandem mass spectrometry (Nano LC-MS/MS). The numbers of protein accession identifiers and their corresponding gene identifiers (gene IDs) obtained from each larvae product samples were compared using Venn diagrams and gene ontology analysis was performed to explore their associated biological processes.
Data source location	INRAE, Université de Tours, BOA, Nouzilly, France.
Data accessibility	Repository name: Recherche Data Gouv Data identification number: doi:10.57745/SBWIJA
Related research article	-

## 1 Value of the Data

• These data provide a comparison of the protein composition of three processed forms of black soldier fly larvae (BSFL), in order to evaluate their protein diversity, similarities, and specificities.
• The data were analyzed using gene ontology terms to give an overview of the potential bioactive proteins and peptides present in BSF larvae.
• Emphasis was placed on the molecules associated with antimicrobial defense and detoxification, as these molecules may play an important role in improving animal gut health.
• The proteomic characterization of BSF larvae is crucial for predicting their value as a source of bioactive components

## 2 Background

Poultry meat production plays a significant role in satisfying global protein needs and is expected to double by 2050 to meet growing demand [1]. However, the poultry industry faces major challenges related to the sustainability of production systems. One strategy is to promote the use of locally available feedstuffs to reduce reliance on imported soybean. The black soldier fly (BSF, *Hermetia illucens*), has emerged as a valuable alternative source of protein [2,3], also known for its natural antibacterial and anticoccidial molecules [[4], [5], [6]]. When provided as larvae, BSF may support gut health in chickens. Nevertheless, one limitation of BSF larvae is their digestibility due to their high chitin content [7,8], while their potential bioactive compounds including proteins remain to be explored.

The main objective of this study was to investigate and compare the protein diversity of three forms of BSF larvae (live and dry larvae, and protein concentrate), using mass spectrometry analysis combined with gene ontology. These data provide insights into the potential soluble bioactive proteins that could be of interest for animal health, in the perspective of incorporating this sustainable source of proteins into poultry diets.

## 3 Data Description

**Fig. 1:**
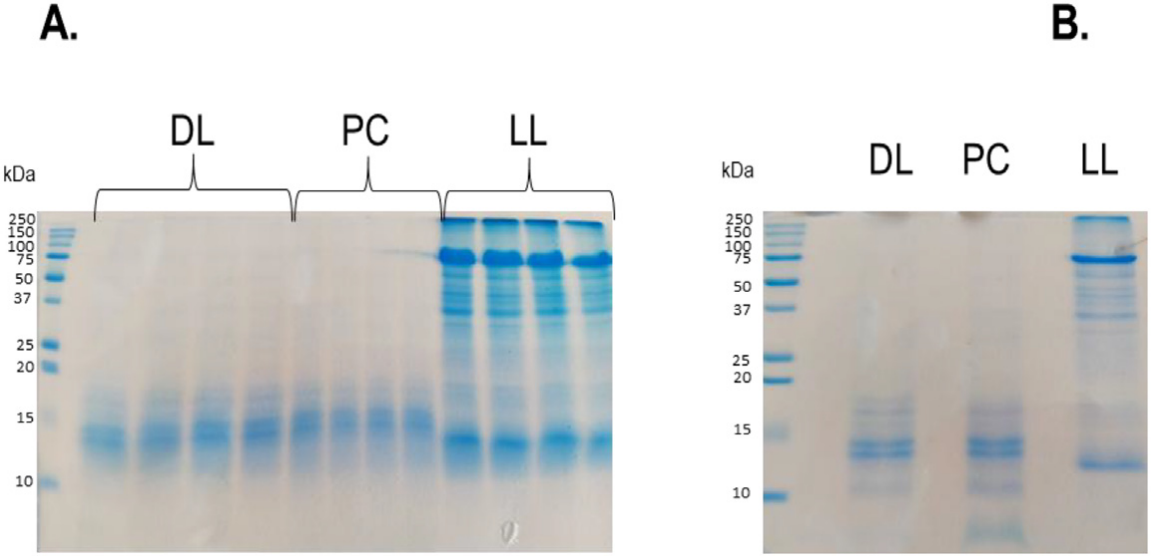
SDS-PAGE analysis of samples (10 µg, 15 % acrylamide-bisacrylamide). A. Individual samples (four technical replicates per type of sample). B. Pooled samples. DL (Dried Larvae); PC (Protein concentrate); LL (Live Larvae).

Protein solubilization from dehydrated larvae (DL), protein concentrate (obtained from dehydrated larvae, PC) and live larvae (LL) was performed as described in the material and methods section. Four technical replicates were obtained for each type of larvae samples, and analyzed by SDS-PAGE followed by ultra-fast staining with Instant blue reagent (Fig. 1A, Material and Methods). The protein profile of the resulting pools (four replicates per type of larvae) was verified by SDS-PAGE followed by R-250 Coomassie blue staining, a classical dye that binds to basic and hydrophobic amino acids and compatible with mass spectrometry analysis (Fig. 1B). The comparison of the results showed that R-250 Coomassie blue staining revealed an additional band below 10 kDa in the PC sample (Fig. 1B). In parallel, the pools were loaded onto a 10 % acrylamide gel, allowed to migrate for 30 min (all proteins combined in a single large band), and stained with R-250 Coomassie blue prior to analysis by mass spectrometry.

The data showed that DL and PC samples mainly contained low molecular weight proteins ranging from 10 to 15 kDa, whereas the LL samples exhibited proteins spanning a much broader range of molecular weights. Noticeably, the PC sample displays an additional low molecular weight band (< 10 kDa) compared with the DL sample, which highlights an enrichment in proteins in this BSFL form.

The raw and filtered data from mass spectrometry analyses are available in an INRAE data repository (10.57745/SBWIJA). The results relating to *Hermetia illucens* species allowed the identification of 1497, 1058 and 2136 distinct proteoforms in PC, DL, and LL, respectively. These numbers correspond to 1463, 1033 and 2136 gene identifiers, respectively (Table 1). However, several proteoforms does not yet have gene identifiers in the EnsemblMetazoa database (https://metazoa.ensembl.org/index.html) (Table 1).

**Table 1:** Summary of the bioinformatic analyses of the mass spectrometry data. DL (Dried Larvae); PC (Protein concentrate); LL (Live Larvae).

	DL	PC	LL
**Number of protein accession identifiers (*Hermetia illucens* species)**	1058	1497	2136
**Number of unique Gene IDs**	1033, including 12 without gene ID (not found)	1463, including 14 without gene ID (not found)	2064, including 15 without gene ID (not found)

The results showed that the number of proteins identified in the LL proteome was significantly higher than that in DL and PC samples. These mass spectrometry results are consistent with our observations from the SDS-PAGE analysis (Fig. 1), which revealed a more complex protein profile for the LL sample compared to DL and PC samples.

**Fig. 2:**
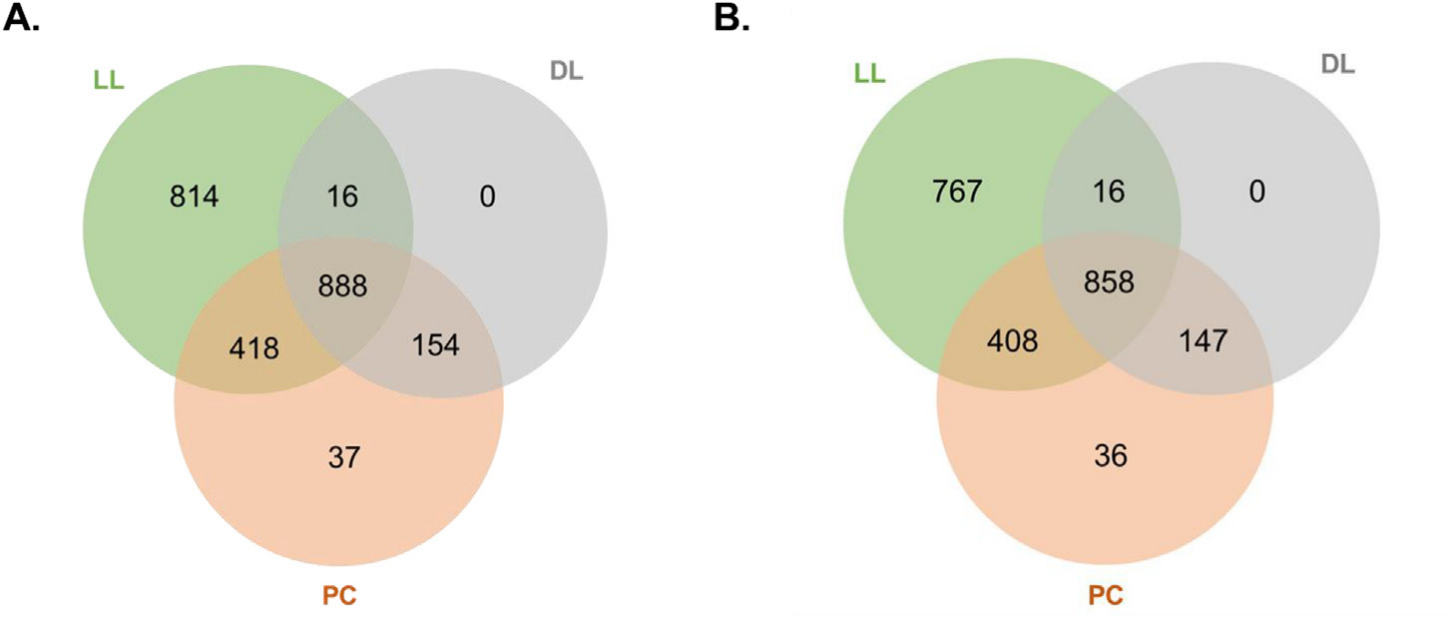
Analysis of shared and sample-specific proteoforms and genes. **A.** Venn diagram comparing proteoforms in larvae samples. **B.** Venn diagram highlighting shared and specific gene IDs excluding “not found” proteins (corresponding to 1021, 1449 and 2049 geneIDs for DL, PC and LL, respectively).

Fig. 2 illustrates the Venn diagrams corresponding to the comparisons of proteoforms and genes identified among the three larvae samples (Fig. 2A and 2B, respectively). The results showed that all three samples shared 888 proteins, which represents 83.9 % of the proteoforms identified in the DL sample and only 59.3 % and 41.5 % of the proteoforms identified in the PC and LL samples, respectively (Fig. 2A). Similar patterns were observed when considering gene IDs: shared gene IDs represented 84.0 %, 59.2 %, and 41.9 % of the genes identified in DL, PC and LL, respectively (Fig. 2B). As shown in Fig. 2, the LL sample is characterized by numerous specific genes and proteins that were not identified in the DL and PC samples, while all molecules identified in the DL sample are common to the LL and/or PC samples.

**Fig. 3:**
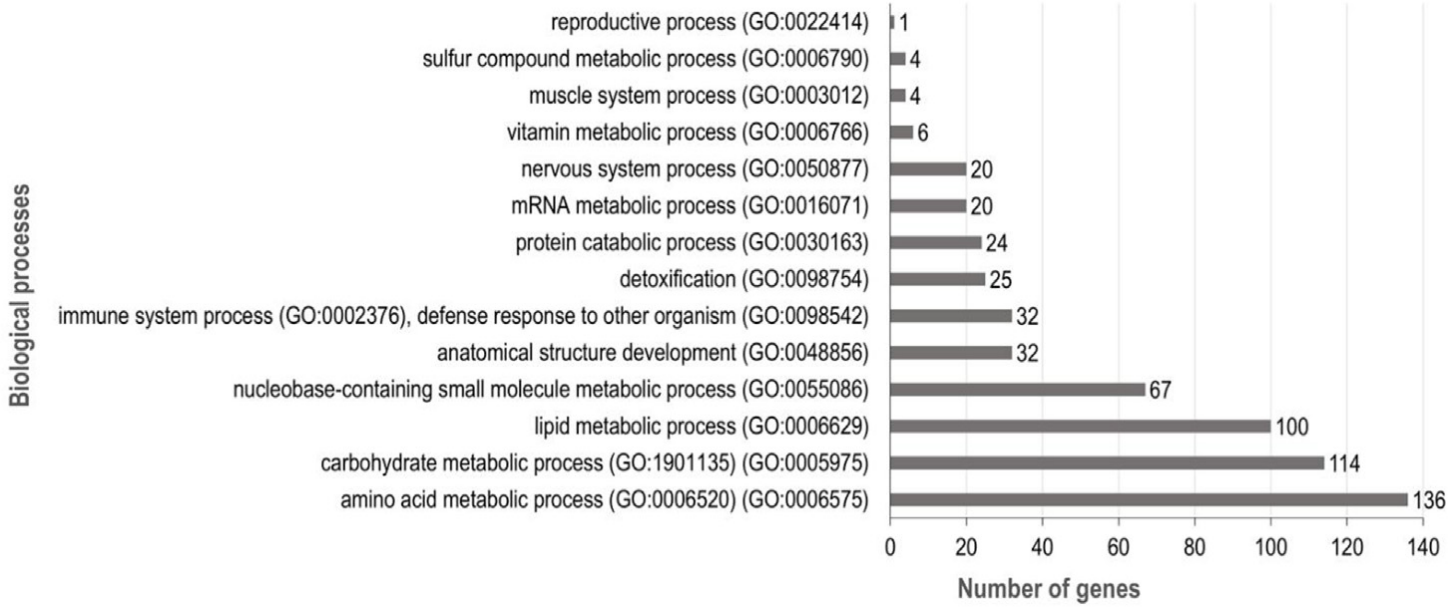
Biological processes associated with proteins found in black soldier fly larvae (Gene ontology analyses).

To assess the potential bioactivity of the soluble proteins identified in larvae, we analyzed their associated biological processes using Biomart from the EnsemblMetazoa database [9]. The analysis was performed using the non-redundant list of gene IDs after compiling data from LL, DL and PC samples (2232 unique gene IDs in total). Among these, 585 proteins were successfully assigned to specific biological processes. Most of these proteins were found to be associated with metabolic processes, including amino acid, carbohydrate, lipid, nucleic acid and vitamin metabolisms (Fig. 3).

Table 2 lists the 57 distinct molecules that could contribute to maintaining gut homeostasis and health of chickens fed black soldier fly larvae (defense response, GO:0098542; immune response, GO:0002376; detoxification, GO:0098754).

**Table 2:** List of proteins associated with immunity and detoxification.

GOSlim GOA Accession(s)	GOSlim GOA Description	Protein name (Gene ID)
**GO:0098542**	**Defense response to other organisms**: Reactions triggered in response to the presence of another organism that act to protect the cell or organism from damage caused by that organism	lysozyme c-1-like (LOC119654068, LOC119654072); lysozyme 2-like (LOC119654086); lysozyme 1-like (LOC119654544); lysozyme-like (LOC119654694); lysozyme 2-like (LOC119655024); invertebrate-type lysozyme 3-like, transcript variant X1 (LOC119655516)
**GO:0002376**	**Immune system process**: Any process involved in the development or functioning of the immune system, an organismal system for calibrated responses to potential internal or invasive threats	gram-negative bacteria-binding protein 1-like (LOC119648301); Attacin_C; Attacin, C-terminal region (LOC119648693); Attacin_C; Attacin, C-terminal region (LOC119648914); gram-negative bacteria-binding protein 3-like (LOC119649581); putative defense protein 2, transcript variant X2 (LOC119651291); Attacin_C; Attacin, C-terminal region (LOC119652481); attacin-A-like (LOC119654856); cecropin-like peptide 1 (LOC119656616, LOC119656617, LOC119656618, LOC119656626); peptidoglycan-recognition protein 2-like (LOC119657102); protein spaetzle-like (LOC119657145); peptidoglycan-recognition protein 1-like, transcript variant X1 (LOC119657197); peptidoglycan-recognition protein SA-like (LOC119657304, LOC119657333); cecropin-like peptide 1 (LOC119657650); protein spaetzle-like, transcript varianLOC119659319); t X1 (LOC119657808); protein spaetzle-like, transcript variant X3 (LOC119658309); defensin-like (LOC119658450); peptidoglycan-recognition protein LB-like (LOC119658694); peptidoglycan-recognition protein LB-like, transcript variant X3 (LOC119660570); peptidoglycan-recognition protein LB-like (LOC119660575, LOC119660576)
**GO:0098754**	**Detoxification**: Any process that reduces or removes the toxicity of a toxic substance. These may include transport of the toxic substance away from sensitive areas and to compartments or complexes whose purpose is the sequestration of the toxic substance	invertebrate-type lysozyme 3-like, transcript variant X1 (LOC119655516); S-formylglutathione hydrolase (LOC119647281); probable phospholipid hydroperoxide glutathione peroxidase, transcript variant X1 (LOC119647472); peroxidasin, transcript variant X2 (LOC119647734); pyrimidodiazepine synthase-like (LOC119648365);peroxiredoxin-6-like (LOC119648772); superoxide dismutase [Cu-Zn] (LOC119649125); peroxiredoxin-1, transcript variant X5 (LOC119649161); copper chaperone for superoxide dismutase (LOC119649624); peroxiredoxin-6-like (LOC119649890); catalase, transcript variant X3 (LOC119649978); peroxiredoxin-6-like (LOC119650213); peroxiredoxin-6, transcript variant X1 (LOC119650708); peroxiredoxin-2, transcript variant X1 (LOC119652357); extracellular superoxide dismutase [Cu-Zn] (LOC119654891); probable cytochrome P450 6g2, transcript variant X1 (LOC119655376); superoxide dismutase [Mn], mitochondrial (LOC119655657); alcohol dehydrogenase class-3 (LOC119656700); alcohol dehydrogenase class-3-like, transcript variant X1 (LOC119657691); alcohol dehydrogenase class-3-like (LOC119657794, LOC119658051); thioredoxin reductase 1, mitochondrial-like, transcript variant X1 (LOC119658295); uncharacterized LOC119659737 (LOC119659737); peroxidase (LOC119659891); peroxiredoxin-5, mitochondrial (LOC119659932); peroxiredoxin 1 (LOC119660964)

Many molecules involved in defense and immune responses are antimicrobial proteins and peptides (lysozymes, attacin-like proteins, defensins, cecropins, peptidoglycan-recognition proteins), while the detoxification process includes numerous peroxiredoxins, peroxidases, dismutases, and alcohol dehydrogenases.

Altogether, these data offer an overview of the proteins composing the water-soluble fraction of BSF larvae and give insight into their natural potential functions and activities.

## 4 Experimental Design, Materials and Methods

### 4.1 Sampling

**Fig. 4:**
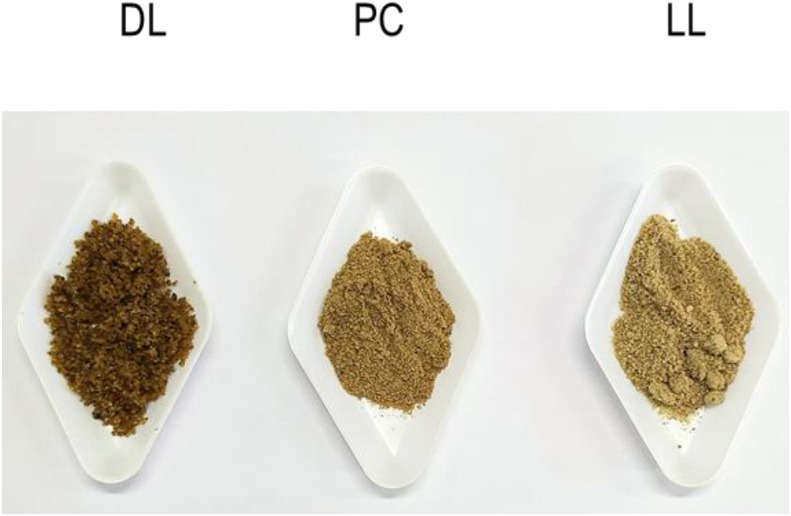
Dried larvae (DL), protein concentrate from dried larvae (PC), and live larvae (LL) after grinding.

*Hermetia illucens* larvae were produced by MUTATEC (Cavaillon, France). The live larvae (LL) were reared on a mixture of plant by-products, including cereals, fruits, and vegetables. A portion of the live larvae was dried to produce DL, while the Protein Concentrate (PC) was produced by the mechanical defatting process of the DL. LL, DL and PC, were ground in the INRAE laboratory (Fig. 4), and then kept at −20 °C until the solubilization process.

### 4.2 Chemicals

All chemicals were from analytical grade.

### 4.3 Sample solubilization and preparation

LL, DL and PC samples (approximately 200 mg of each) were solubilized in 1 mL 50 mM Tris–HCl, 1 M NaCl, pH 7.4, and then ground using a mechanical ball milling for 5 min at 30 Hz. Four replicates per sample were performed for each sample (12 samples in total). The samples were centrifuged for 10 min at 21,130 rpm at 4 °C. The supernatant was then filtered through a 0.45 µm filter to remove insoluble aggregates. The protein concentration of each sample was estimated using absorbance at 280 nm and the samples were diluted to determine protein concentration using DC Biorad reagent (Biorad, Marne-la-Coquette, France) and a standard curve (bovine serum albumin, 0–2 mg).

### 4.4 SDS-PAGE analysis of replicates

To evaluate the reproducibility of the preparations of each form of larvae, individual supernatants (10 µg) were diluted in 5X Laemmli buffer under non-reducing conditions, then boiled, subjected to electrophoresis on a 15 % acrylamide/bisacrylamide gel, and stained with Instant blue (Sigma-Aldrich, Saint-Quentin-Fallavier, France). After verifying the homogeneity of the SDS-PAGE profiles, a pool of the three technical replicates per sample type (LL, DL and PE) was made. One µg of each pool was diluted in 5X Laemmli buffer, boiled, deposited onto a 15 % acrylamide/bisacrylamide gel, and subjected to migration, as described above. In parallel, about 35 µg of the pools were deposited onto a 10 % acrylamide/bisacrylamide gel and subjected to migration at 50 V for 20 min in order that the proteins concentrate in one single large band (after R-250 Coomassie blue staining). The bands from each of the three pools were cut, and kept at −20 °C prior to mass spectrometry analysis.

### 4.5 Mass spectrometry analysis

#### 4.5.1 Sample preparation

Each gel slice was rinsed separately in water and then acetonitrile. The proteins were then reduced with dithiothreitol, alkylated with iodoacetamide, and digested with trypsin, as previously described [10]. The extracted peptides were dried prior to MS analysis. Dried peptides were re-suspended in 120 µL buffer A (100 % water in presence of 0.1 % formic acid, Sigma) and sonicated for 10 min. Aliquots (20 μL) were loaded onto EvoTip Pure tips (Evosep One, Odense, Denmark) according to the manufacturer’s instructions. The peptides were separated on a 15 cm C18 Endurance analytical LC column (1.9 µm ReproSil Saphir C18 beads, ID 150 µm x 15 cm, EV-1106) using the standardized 15 samples-per-day (SPD) Extended method. The gradient was obtained by flow injection of the solvent A (100 % H2O acidified with 0.1 % formic acid, Sigma) and solvent B (100 % acetonitrile acidified with 0.1 % formic acid, Sigma-Aldrich). Peptides were eluted, at a flow rate of 220 nL/min, by a linear gradient up to 35 % solvent B. The column was connected to a 20 µm ID fused silica emitter (EV 1087). The Evosep column was mounted on a nano-spray Flex NG™ source (Thermo Fisher Scientific, Bremen, Germany).

#### 4.5.2 NanoLC-MS/MS

The Tribrid Orbitrap Ascend mass spectrometer (Thermo Fisher Scientific, Bremen, Germany) was operated in Data Dependent Acquisition and in positive ion modes, automatically switching between MS and MS/MS acquisition for a 3 s cycle time. The source voltage was 1.9 kV, the capillary temperature was set at 275 °C and S-lens at 60 %. Full-scan MS profile spectra were acquired from 350 to 1400 *m/z* mass range in the Orbitrap. Resolution in the Orbitrap analyzer was set at *R* = 240,000 (at *m/z* 200). Monoisotopic peak determination (MIPS) was carried out in peptide mode on the most abundant peaks, presenting two to seven charges and a minimum intensity threshold at 5.103. The precursor ions were filtered using the quadrupole with an isolation window of 1.6 *m/z* and isolated in the linear ion trap. In the high-pressure cell of the ion trap, higher energy collisional dissociation fragmentation mode was carried out with a fixed normalized collision energy at 30 %. MS2 centroid spectra (1 microscan) were obtained using the Rapid ion trap scan rate ranged from 200–1400 *m/z*. Dynamic exclusion was activated for 60 s with a repeat count of one, using a mass tolerance of 10 ppm, excluding the corresponding isotopes. A lock mass correction enabled for accurate mass measurements using the run-start Easy-IC™ mode using fluoranthene cations (at 202.07770 *m/z*).

For all experiments, the FAIMS Pro Duo interface (Thermo Fisher Scientific, San Jose, CA) was set to the standard resolution using a nitrogen carrier gas set at 4.0 L/min. For each sample, the proteomic experiments were performed in duplicates using two different MS methods with three constant compensation voltages (CVs), by nanoLC-FAIMS-MS2. Ion fractionation was applied using the combination −35, −50, −70 CVs or −45, −60, −75 CVs, allowing transmission of specific groups of ions for 1.2 *sec*, 1 *sec* and 0.8 *sec*, respectively.

The raw data were submitted for analysis in Proteome Discoverer™ (PD) 3.1.1.93 (Thermo Fisher Scientific) software for assignment of MS/MS spectra using the Chimerys 2.0 (MSAID) algorithm [11]. The top-20 most abundant peaks were filtered within 100 Da mass window. The following parameter settings have been used for searches: trypsin as the enzyme, two missed cleavages, peptide length of 7–30 residues, and a MS2 fragment mass tolerance of 0.3. Carbamidomethyl on cysteine amino acids (+57.021464 Da) was set as a static modification, while methionine oxidation (+15.99492 Da) was set as a variable modification. Peptide spectrum matches were achieved using UniprotKB protein database (http://www.uniprot.org/) [12]. All 17 607 entries from *Hermetia illucens* taxon (sp_tr_canonical TaxID = 343,691_and_subtaxonomies) (v2024–10–02) were downloaded. The results obtained from the target and decoy databases searches were retained using a False Discovery Rate < 1 % at the peptide and protein levels. Protein identifications were validated if the proteins identified had a high confidence score with a q-value < 0.05, even if they contained only one identified peptide.

#### 4.5.3 Gene ontology analysis

A list of proteins combining protein accession numbers from all three samples was generated. From this list, the unique gene IDs were then retrieved using Biomart from the EnsemblMetazoa database (https://metazoa.ensembl.org/index.html) and *Hermetia illucens* dataset (iHerIll2.2.curated.20191125). The filter used was “gene stable IDs” and the attributes included “Gene stable ID”, “GOSLim GOA accession”, and “GOSlim GOA Description”. From this list, only GO terms relating to biological processes were used (https://doi.org/10.57745/SBWIJA).

## Limitations

SDS-PAGE profiles and mass spectrometry results may vary depending on several factors, including the developmental stage of the larvae [13], the substrate used for larval rearing [14], the technological process [15], and the experimental procedure used to obtain the soluble fraction of proteins. It is noteworthy that the non-denaturing process used to solubilize the larvae is a mild process, which does not enable the extraction of insoluble proteins or aggregates. Therefore, the list of proteins provided in this work is not exhaustive but rather highlights proteins that are likely to be easily solubilized during the digestion in chickens and thus susceptible to be active (not denatured), and play a role in gut health. It is noteworthy that this article is descriptive and does not draw any conclusions about the efficacy of these components, which must be further validated by in vitro activity tests and in vivo experiments.

## Ethics Statement

The authors have read, and follow the ethical requirements for publication in Data in Brief. The authors confirm that the current work does not involve human subjects, animal experiments, or any data collected from social media platforms.

## Credit Author Statement

**Abdel-Moneim Eid Abdel-Moneim:** Investigation, Methodology, Writing - Review & Editing. **Daniel Tomas:** Investigation, Formal Analysis, Data Curation, Writing - Review & Editing. **Valérie Labas:** Validation, Data Curation, Writing - Review & Editing. **Bertrand Méda:** Project administration, resources, Writing - Review & Editing. **Sophie Réhault-Godbert:** Project administration, Conceptualization, Methodology, Supervision, Writing - Visualization, Original Draft, Review & Editing.

## Data Availability

DataverseSupporting data relating to the proteomes of three water-soluble extracts from Hermetia illucens larvae (Original data).

## References

[1] FAO (2018).

[2] Lu S., Taethaisong N., Meethip W., Surakhunthod J., Sinpru B., Sroichak T., Archa P., Thongpea S., Paengkoum S., Purba R.A.P., Paengkoum P. (2022). Nutritional composition of black soldier fly larvae (Hermetia illucens L.) and its potential uses as alternative protein sources in animal diets: a review. Insects.

[3] Belhadj Slimen I., Yerou H., Ben Larbi M., M'Hamdi N., Najar T. (2023). Insects as an alternative protein source for poultry nutrition: a review. Front. Veterin. Sci..

[4] Park S.I., Kim J.W., Yoe S.M. (2015). Purification and characterization of a novel antibacterial peptide from black soldier fly (Hermetia illucens) larvae. Dev. Comp. Immunol..

[5] Franco A., Scieuzo C., Salvia R., Pucciarelli V., Borrelli L., Addeo N.F., Bovera F., Laginestra A., Schmitt E., Falabella P. (2024). Antimicrobial activity of lipids extracted from Hermetia illucens reared on different substrates. Appl. Microbiol. Biotechnol..

[6] Sedano L., Abert Vian M., Guidou C., Bussière F.I., Lacroix-Lamandé S., Trespeuch C., Méda B., Silvestre A. (2025). Research note: in vitro anticoccidial activity of protein and lipid extracts from the black soldier fly larvae (Hermetia illucens). Poult. Sci..

[7] Bonomini M.G., Prandi B., Caligiani A. (2024). Black soldier fly (Hermetia illucens L.) whole and fractionated larvae: in vitro protein digestibility and effect of lipid and chitin removal. Food Res. Int..

[8] Schiavone A., De Marco M., Martínez S., Dabbou S., Renna M., Madrid J., Hernandez F., Rotolo L., Costa P., Gai F., Gasco L. (2017). Nutritional value of a partially defatted and a highly defatted black soldier fly larvae (Hermetia illucens L.) meal for broiler chickens: apparent nutrient digestibility, apparent metabolizable energy and apparent ileal amino acid digestibility. J. Anim. Sci. Biotechnol..

[9] Yates A.D., Allen J., Amode R.M., Azov A.G., Barba M., Becerra A., Bhai J., Campbell L.I., Carbajo Martinez M., Chakiachvili M., Chougule K., Christensen M., Contreras-Moreira B., Cuzick A., Da Rin Fioretto L., Davis P., De Silva N.H., Diamantakis S., Dyer S., Elser J., Filippi C.V., Gall A., Grigoriadis D., Guijarro-Clarke C., Gupta P., Hammond-Kosack K.E., Howe K.L., Jaiswal P., Kaikala V., Kumar V., Kumari S., Langridge N., Le T., Luypaert M., Maslen G.L., Maurel T., Moore B., Muffato M., Mushtaq A., Naamati G., Naithani S., Olson A., Parker A., Paulini M., Pedro H., Perry E., Preece J., Quinton-Tulloch M., Rodgers F., Rosello M., Ruffier M., Seager J., Sitnik V., Szpak M., Tate J., Tello-Ruiz M.K., Trevanion S.J., Urban M., Ware D., Wei S., Williams G., Winterbottom A., Zarowiecki M., Finn R.D., Flicek P. (2021). Ensembl Genomes 2022: an expanding genome resource for non-vertebrates. Nucleic Acids Res..

[10] Labas V., Grasseau I., Cahier K., Gargaros A., Harichaux G., Teixeira-Gomes A.P., Alves S., Bourin M., Gérard N., Blesbois E. (2015). Qualitative and quantitative peptidomic and proteomic approaches to phenotyping chicken semen. J. Proteom..

[11] Dorfer V., Maltsev S., Winkler S., Mechtler K. (2018). CharmeRT: boosting peptide identifications by chimeric spectra identification and retention time prediction. J. Proteome Res..

[12] Consortium T.U. (2024). UniProt: the Universal protein knowledgebase in 2025. Nucleic Acids Res..

[13] Khan J.A., Guo X., Pichner R., Aganovic K., Heinz V., Hollah C., Miert S.V., Verheyen G.R., Juadjur A., Rehman K.U. (2025). Evaluation of nutritional and techno-functional aspects of black soldier fly high-protein extracts in different developmental stages. Animal.

[14] Zandi-Sohani N., Tomberlin J.K. (2024). Comparison of growth and composition of black soldier fly (Hermetia illucens L.) larvae reared on sugarcane by-products and other substrates. Insects.

[15] Ravi H.K., Degrou A., Costil J., Trespeuch C., Chemat F., Vian M.A. (2020). Effect of devitalization techniques on the lipid, protein, antioxidant, and chitin fractions of black soldier fly (Hermetia illucens) larvae. Europ. Food Res. Technol..

